# Oral Carnosine Supplementation Preserves Vascular Function of Sprague Dawley Rats on a High-Salt Diet via Restored Antioxidative Defence

**DOI:** 10.3390/nu17010036

**Published:** 2024-12-26

**Authors:** Ines Drenjančević, Ana Stupin, Ivana Jukić, Nikolina Kolobarić, Petar Šušnjara, Nataša Kozina, Lora Kovač, Zrinka Mihaljević

**Affiliations:** 1 Department of Physiology and Immunology, Faculty of Medicine Osijek, Josip Juraj Strossmayer University of Osijek, J. Huttlera 4, 31000 Osijek, Croatia; ines.drenjancevic@mefos.hr (I.D.); ana.stupin@mefos.hr (A.S.); ivana.jukic@mefos.hr (I.J.); nbdujmusic@mefos.hr (N.K.); natasa.kozina@mefos.hr (N.K.);; 2 Scientific Center of Excellence for Personalized Health Care, Josip Juraj Strossmayer University of Osijek, Trg Svetog Trojstva 3, 31000 Osijek, Croatia; 3 Faculty of Kinesiology Osijek, Josip Juraj Strossmayer University of Osijek, 31000 Osijek, Croatia; psusnjara@kifos.hr

**Keywords:** high-salt diet, carnosine, isolated aortic rings, hypoxia-induced relaxation, Sprague Dawley rats

## Abstract

**Backgrounds/Objectives**: Following previous findings on high-salt (HS)-intake-related increase of oxidative stress, this study explored whether carnosine (CAR; β-alanyl-L-histidine), a reactive oxygen species (ROS) scavenger, enhanced antioxidative defence and vascular function following HS, potentially via the NRF2 or HIF-1α signalling pathway. **Methods**: Sprague Dawley rats (64, 8–10 weeks old, both sexes) were divided into four groups (n = 6/group): CTRL (0.4% NaCl), HS (4% NaCl for 7 days), CTRL + CAR (0.4% NaCl and 150 mg/kg/day oral CAR supplementation), and HS + CAR (4% NaCl and CAR). Acetylcholine-induced relaxation (AChIR) and hypoxia-induced relaxation (HIR) were evaluated in norepinephrine-precontracted (NE, 10^−7^ M) aortic rings. HIR was also tested with NRF2 (ML-385, 5 × 10^−6^ M) and HIF-1α (LW6, 10^−4^ M) inhibitors. Gene expression of superoxide dismutases 1, 2, and 3 (*SOD1, 2* and *3*), glutathione peroxidases (*GPx1* and *4)*, catalase (*CAT*), *NRF2*, and NAD(P)H dehydrogenase (quinone 1) (*NQO1*) in aortic tissue was measured by RT-qPCR. Ferric reducing antioxidant power (FRAP) and advanced oxidation protein products (AOPPs) assays were performed on serum samples. All experimental procedures conformed to the European Guidelines (directive 86/609) and were approved by the local and national Ethical Committees (#2158-61-46-23-36, EP355/2022). **Results:** HS impaired AChIR and HIR, both preserved by CAR. NRF2 and HIF-1α inhibitors suppressed HIR in the HS and HS + CAR groups. CAR significantly increased *SOD1* and *2*, *NRF2*, and *NQO1* expression and SOD activity compared to the CTRL and HS groups. *GPx1* and *GPx4* were upregulated in HS + CAR compared to HS. CAR prevented an increase in AOPPs, which were elevated in HS, while FRAP was highest in HS + CAR. **Conclusions:** Carnosine enhances antioxidative defence by upregulating antioxidant enzymes and activities and preserves vascular relaxation, likely via NRF2 signalling.

## 1. Introduction

Over the past 30 years, substantial evidence has emerged indicating that a high dietary intake of kitchen salt (sodium chloride, NaCl)—commonly referred to as a high-salt (HS) diet—is an independent risk factor for the development of cardiovascular diseases. This effect is distinct from its well-recognized role in raising arterial pressure in certain individuals. Studies involving normotensive experimental animals and humans have demonstrated that a critical aspect of this pressure-independent effect of dietary salt is the reduction in vascular nitric oxide (NO) bioavailability, which impairs endothelium-dependent dilation. This reduction in NO is closely linked to elevated levels of reactive oxygen species (ROS) produced by NAD(P)H oxidase, xanthine oxidase, or uncoupled endothelial NO synthase in the vascular wall. These ROS not only scavenge NO but also disrupt signalling pathways crucial for its synthesis [1,2,3]. Endothelial function is widely recognized as being adversely affected by increased oxidative stress, which impairs vasoreactivity and fosters pro-inflammatory, pro-atherogenic, and pro-thrombotic conditions that contribute to atherosclerosis, hypertension, and other cardiometabolic disorders. Treatment with superoxide dismutase or TEMPOL, a superoxide scavenger, has been shown to restore vasodilation and NO levels to those observed in animals on a normal-salt diet, confirming the role of superoxide (O_2_^•−^) in NO oxidation under these conditions [4,5]. In our previous work, we demonstrated that an HS diet induces changes in vascular reactivity by increasing vascular and systemic oxidative stress and altering flow-induced dilation (FID) mechanisms. Specifically, while FID in animals on a low-salt (LS) diet was mediated by NO, prostanoids, and epoxyeicosatrienoic acids (EETs), FID in animals on an HS diet was attenuated and entirely dependent on NO [5].

Carnosine (CAR; β-alanyl-L-histidine) is a dipeptide that has anti-inflammatory and antioxidant activity, and acts as a “scavenger” of reactive oxygen species (ROS) directly by donating hydrogen [6]. It is known that carnosine reduces peroxidation of lipids and low-density lipoprotein in the serum of aged rats [7]. Carnosine has the ability to chelate transition metals and inhibits carbonylation, glycosylation, and aggregation of proteins [8,9,10,11]. This effect of carnosine may be important, having in mind that increased oxidative stress leads to production of oxidized LDL (oxLDL) and advanced oxidized protein products (AOPPs), which have been shown to enhance endothelial injury [12,13]. To our knowledge, there are no studies that have examined the mechanistic effect of carnosine on vascular function and oxidative stress levels following HS intake.

Indirectly, carnosine activates the nuclear factor erythroid 2-related factor 2 (NRF2) signalling pathway, which promotes the expression of genes encoding antioxidant enzymes, providing protection against oxidative damage [9,10,14,15,16]. Upon activation, NRF2 translocates to the nucleus and binds to antioxidant response elements (AREs) in the promoter regions of target genes, resulting in the upregulation of antioxidant enzymes such as heme oxygenase-1 (HO-1) and superoxide dismutase (SOD) [17]. Compared to exogenous antioxidant supplementation, NRF2 activation offers more robust antioxidative effects, making it a promising therapeutic target for oxidative stress-related diseases [18]. NRF2 has several target genes that are involved in antioxidative cellular defence. For example, induction of NAD(P)H dehydrogenase [quinone] 1 (NQO1) is mediated through the Keap1/Nrf2/ARE pathway [18,19,20]. NQO1 contributes to cellular protection against oxidative stress by detoxifying vitamin K3 and participating in the metabolism of ubiquinone and vitamin E quinone [19]. Other antioxidative enzymes, such as SOD, CAT, and GPx are also target genes of NRF2 [17].

The transcription factor hypoxia-inducible factor alpha (HIF-1α) and its target genes have been suggested to be involved in mechanisms of vasodilation that were altered by HS intake [2,5]. Interestingly, the interplay between HIF-1α and NRF2 involves multiple mechanisms. NRF2 silencing has been shown to decrease HIF-1α levels, suppressing HIF-1α-driven processes such as cell proliferation, angiogenesis, tumor growth, and migration/invasion [21]. On the other hand, NRF2 activation has increased HIF-1α expression [22]. Additionally, NRF2 target genes, such as *NQO1*, contribute to the stabilization of HIF-1α. Conversely, HIF-1α activation can suppress NRF2 transcriptional activity, as demonstrated in ischemic mouse kidneys and vascular endothelial cells [22,23]. Previously, it has been shown that hypoxia-induced relaxation was impaired by an HS diet [4] and could be restored by TEMPOL, a superoxide scavenger [24]. Oxidative stress stimulates HIF activation, further influencing the interaction of NRF2 and HIF-1α. These findings indicate that hypoxia and oxidative stress are interconnected rather than independent factors.

Considering that an HS diet induces increased oxidative stress, leading to impaired vascular reactivity, and that carnosine has antioxidative effects, the hypothesis of the present study was that concomitant carnosine supplementation to HS diet will reduce oxidative stress induced by HS intake and consequently preserve vascular relaxation in Sprague Dawley rats. Furthermore, that the effect of carnosine occurs via the NRF2 and/or HIF-1α pathways.

This study aimed to investigate the effect of carnosine supplementation on macrovascular function by examining acetylcholine-induced and hypoxia-induced vasorelaxation of isolated aortic rings and different biomarkers of oxidative stress (i.e., AOPP, FRAP) as well as expression of antioxidative enzymes and enzymes producing mediators of vascular relaxation, expression of transcription factors *HIF-1α* and *NRF2,* and activity of SOD in Sprague Dawley rats on an HS diet.

## 2. Materials and Methods

### 2.1. Ethical Approval

The experimental procedures were approved by Ethical Committees (Faculty of Medicine, University of Osijek; class: 602-04/22-08/07, no. 2158-61-46-22-14, 2 February 2022; National Ethical Committee for the Protection of Animals Used for Scientific Purposes EPP355/2022, 23 March 2022; and Ministry of Agriculture, Croatia: class: UP/I-322-01/22-01/9, no. 525-09/566-22-3, 14 July 2022), adhering to European Guidelines for the Care and Use of Laboratory Animals (Directive 2010/63/EU).

### 2.2. Experimental Animals and Carnosine Supplementation Protocol

Sixty-four Sprague Dawley (SD) rats (both sexes, 8–10 weeks old) were housed at the Faculty of Medicine Osijek, Croatia, at the registered and certified animal care facility (HR-POK-005) at standardized conditions, at a temperature of 21 °C–23 °C, a humidity- and light-controlled room with free access to tap water, and fed ad libitum with a commercially prepared pellet diet (Mucedola, Milan, Italy). The rats were randomly divided into four groups (6 rats/per group): CTRL (control group, 0.4% NaCl), HS group (rats fed 4% NaCl in the rat chow for 7 days), CTRL + CAR group (rats administered oral carnosine supplementation, 150 mg/kg/day for 7 days by oral gavage), and HS + CAR group (rats fed HS diet and receiving oral carnosine supplementation). The dose of carnosine was determined by reviewing the literature [25]. Body mass and arterial pressure (systolic blood pressure, diastolic blood pressure and mean arterial pressure) were analysed for each sex separately and as group values, while all other results were pulled and analysed as a group without division by sex of the animals.

### 2.3. Blood Pressure Measurement and Blood Sampling

On the day of the experiment and prior to arterial blood pressure (ABP) measurements, rats were anesthetized with a combination of ketamine (75 mg/kg) (Ketanest S 25 mg/mL, Pfizer Pharma GmbH, Berlin, Germany) and midazolam (0.5 mg/kg) (Midazolam Torrex 5 mg/mL, Torrex Chiesi Pharma, Parma, Italy). ABP was measured with the Edan X10 (Edan Instruments Inc., Shanghai International Holding Corp. GmbH, Hamburg, Germany) using a PE-50 catheter inserted into the left femoral artery according to the established protocol described previously [26,27]. Briefly, after cannulation of the femoral artery, and 10 min of stabilization, systolic and diastolic ABP were determined as the mean value of recorded ABP every 10 s within 1 min of measurement. The results are presented as mean arterial pressure (MAP).

Upon ABP measurements, anesthetized animals were decapitated by guillotine, blood was drawn from the same animals to obtain serum samples; next, aorta samples were taken and frozen in liquid nitrogen immediately after isolation and then stored at −80 °C until further analyses.

### 2.4. Assessment of Vasorelaxation of Aortic Rings 

The experiments involving aortic rings were conducted following the established protocol in our laboratory [27,28,29,30,31]. A separate set of rats (n = 5 per group) divided into the named groups was anesthetized as for the blood pressure measurements and decapitated. After decapitation, the descending thoracic aorta was carefully freed from surrounding connective tissue and placed in an oxygenated modified Krebs–Henseleit solution, then sectioned into rings approximately 3–4 mm in length. The Krebs–Henseleit solution comprised the following (mM): NaCl, 113; KCl, 4.7; MgSO_4_·6H_2_O, 1.2; NaHCO_3_, 22.0; CaCl_2_·2H_2_O, 1.3; KH_2_PO_4_, 1.2; EDTA, 0.026; and glucose, 11.1. During the experiment, this solution was continuously bubbled with a gas mixture of 95% O_2_ and 5% CO_2_ and maintained at a temperature of 37 °C. Each aortic ring was fitted with two stainless-steel hooks inserted into its lumen, and then mounted on a holder placed in a 10 mL organ bath. The upper hook was connected to the arm of a transducer via a filament. A basal resting tension of 2.0 g was applied to each aortic ring, allowing for a 60 min equilibration period, during which the Krebs–Henseleit solution was replaced with fresh solution every 15 min, and passive tension was readjusted to 2.0 g as necessary. To assess the integrity of the endothelium, the rings were precontracted with 10^−7^ M norepinephrine (NE, final concentration), allowed to stabilize for 5 min, and then relaxation was induced using 10^−5^ M acetylcholine. Rings that did not exhibit at least 50% of relaxation were excluded from further experiments. Conversely, those that did relax were washed three times with fresh solution and allowed to equilibrate for an additional 30 min, with washing occurring every 10 min. Subsequently, the response of the aortic rings to acetylcholine (ACh) in cumulative doses (ranging from 10^−9^ to 10^−5^ M) was assessed. Additionally, relaxation in response to reduced pO_2_ (hypoxia-induced relaxation; HIR) was evaluated in a separate set of animals (n = 5 per group). In the HIR experiments, after the equilibration and recovery phases, the rings were precontracted with norepinephrine (NE) at a concentration of 10^−7^ M. The gas mixture was then altered from 95% O_2_ and 5% CO_2_ to 0% O_2_ with 5% CO_2_ for a duration of 20 min, after which it was switched back to the original mixture for 5 min to facilitate reoxygenation. HIR was assessed with/without the NRF2-specific inhibitor ML-385 (N-[4-[2,3-dihydro-1-(2-methylbenzoyl)-1H-indol-5-yl]-5-methyl-2-thiazolyl]-1,3-benzodioxole-5-acetamide, Cayman Chemicals, Ann Arbor, Michigan, USA, 5 × 10^−6^ M) and the HIF-1 alpha-specific inhibitor LW-6 (3-(2-(4-Adamantan-1-yl-phenoxy)-acetylamino)-4-hydroxybenzoic acid methyl ester, Calbiochem, San Diego, CA, USA, 10^−4^ M). The degree of relaxation was quantified as the percentage reduction in NE-induced vasoconstriction. Additionally, the sensitivity of the smooth vascular muscle to nitric oxide (NO) was assessed using intact rings from all experimental groups. The aortic rings were subjected to cumulative doses of sodium nitroprusside dihydrate (SNP, ranging from 10^−10^ to 10^−4^ M), which acts as an endothelium-independent NO donor.

### 2.5. Aortic Tissue mRNA Expression Analyses

Total RNA was extracted from the isolated aortas using the TRIZOL reagent (Invitrogen, Carlsbad, CA, USA) following the manufacturer’s protocol and a protocol established in our laboratory [2,5,13,32]. Purification of the RNA samples and synthesis of complementary DNA (cDNA) was carried out according to the instructions provided by Sigma-Aldrich (Darmstadt, Germany) and Applied Biosystems (Carlsbad, CA, USA), respectively. The concentration and purity of the RNA were determined using a nanophotometer P300 UV–vis (Implen, Munich, Germany). The synthesized cDNA was diluted five-fold in nuclease-free water for real-time PCR analysis. The gene expression analysis was performed on a Bio-Rad CFX96 real-time PCR detection system. The primers used for gene expression analysis, including the nucleotide sequences, are listed in Table 1. The results are presented as relative expression of a particular gene quantified in relation to the expression of the house-keeping gene β-actin.

### 2.6. Advanced Oxidation Protein Products (AOPPs) and Ferric Reducing Antioxidant Power (FRAP) Assays

A commercially available ELISA kit was used to determine AOPPs (MyBioSource, MBS930313, San Diego, CA, USA) in serum samples according to the manufacturer’s instructions. Serum samples were obtained from the blood collected after decapitation and centrifuged for 10 min at 3500 RPM. The antioxidant capacity was measured by the Ferric Reducing Antioxidant Power (FRAP) assay. In this assay, antioxidants were evaluated as reductants of Fe^3+^ to Fe^2+^, which was chelated by TPTZ (2,4,6-Tris(2-pyridyl)-s-triazine) to form an Fe^2+^–TPTZ complex absorbing at 593 nm [33]. The results were compared with a standard curve of Trolox, a water-soluble analogue of Vitamin E, and expressed as mM Trolox equivalents.

### 2.7. Superoxide Dismutase (SOD) Enzyme Activity Assay

Superoxide dismutase (SOD) activity in serum samples was determined following the protocol established and previously published by Cosic et al. [2] and determined based on its ability to inhibit the reduction of cytochrome C by superoxide anions, using xanthine and xanthine oxidase in the reaction. This activity was measured following a modified method outlined by Flohé and Ötting [34]. Calibration was performed using known amounts of purified bovine SOD. An enzyme activity assay was conducted using a Lambda 25 UV–vis spectrophotometer equipped with the UV WinLab 6.0 software package (PerkinElmer for the Better, Waltham, MA, USA) and the results were expressed in units per milligram of protein (U/mg P). The concentration of proteins in the serum samples (mg/mL) was determined according to the manufacturer’s protocol for the Bradford reagent at 595 nm (Bradford Reagent B6916, Sigma Aldrich, St. Louis, MO, USA), using bovine serum albumin as a standard.

### 2.8. Statistical Analysis

The results are presented as arithmetic means (standard deviation; SD), and *p* < 0.05 was considered statistically significant. A two-way ANOVA test was used to test differences in ACh-induced dilation followed by the Tukey post hoc test and one-way ANOVA followed by Holm–Sidak or Kruskal–Wallis’s test to test the difference in response to hypoxia and SNP among the groups. One-way ANOVA was also used to test differences in body mass, ABP, and RT-qPCRs. GraphPadPrism, Version 6.01 for Windows (GraphPad Software, Boston, MA USA), was used for statistical analysis and graphical presentation of the results.

## 3. Results

### 3.1. Body Mass and Blood Pressure of Studied Groups

The mean body mass (g) of rats and MAP (mmHg) were not significantly different among the examined groups (Table 2). Male rats had significantly larger body mass, systolic blood pressure, and MAP compared to female rats, which reflects physiological differences and is within the physiological range [35]. HS intake or carnosine supplementation did not influence body mass or arterial blood pressure values. There were no differences in body mass or arterial pressure between experimental groups for males or females.

### 3.2. Effect of Carnosine Supplementation on Acetylcholine-Induced Relaxation (AChIR)

**Figure 1:**
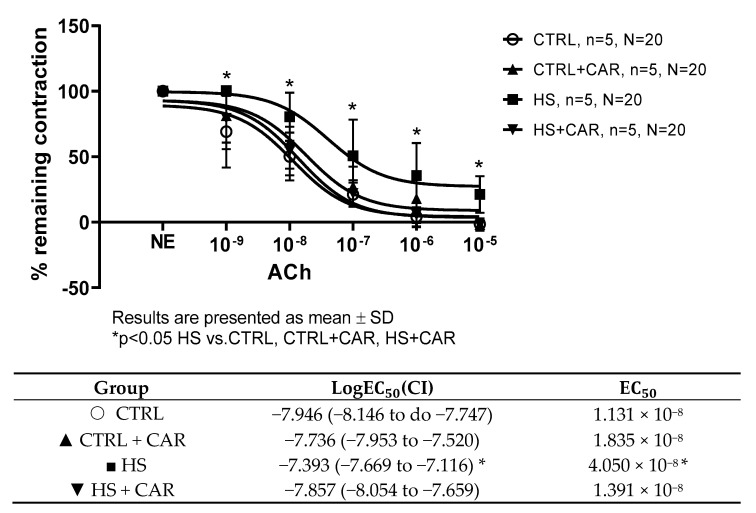
ACh-induced relaxation (AChIR) of isolated rat aorta rings in the CTRL, CTRL + CAR, HS, and HS + CAR groups. The table below the graph presents sensitivity to Ach, represented by logEC50. Results are presented as mean ± SD; n—number of rats; N—number of aortic rings; NE—norepinephrine; * *p* < 0.05, two-way ANOVA test for AChIR, one-way ANOVA test for logEC50 and EC50 values.

Figure 1 shows that ACh at concentrations of 10^−9^ to 10^−5^ M in all four groups of rats induced dilation. However, AChIR of the aortic rings was significantly attenuated in the HS group of rats at all applied doses of ACh compared to all other groups. Additionally, the HS group showed reduced sensitivity to ACh, as indicated by the LogEC_50_ value. The EC_50_ (half maximal effective concentration) represents the concentration of ACh that induces a response halfway between the baseline response and the maximum dilation presented in the table below graph (Figure 1). No difference was observed in the relaxation response to ACh or sensitivity to ACh in the aortas of rats receiving oral carnosine supplementation (CTRL + CAR, HSD + CAR) compared to the CTRL group. A significantly stronger relaxation response to ACh was observed in the aortas of carnosine-treated rats compared to the HS group (Figure 1).

### 3.3. Effect of Carnosine Supplementation on Endothelium-Independent Relaxation Assessed by Sodium Nitroprusside (SNP)

**Figure 2:**
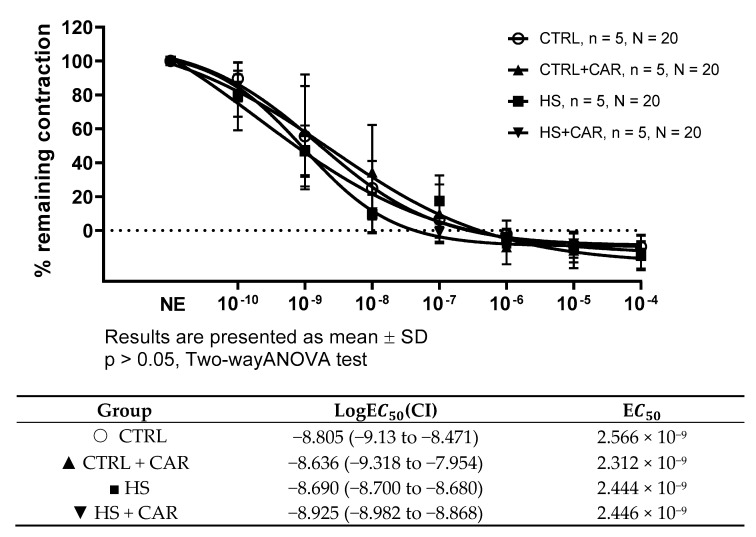
Endothelium-independent dilation of isolated rat aorta rings in the CTRL, CTRL + CAR, HS, and HS + CAR groups assessed by sodium nitroprusside (SNP). Results are presented as mean ± SD; n—number of rats; N—number of aortic rings; NE—norepinephrine; *p* > 0.05, two-way ANOVA test for endothelium-independent dilation, one-way ANOVA test for logEC50 and EC50 values.

To assess the endothelium-independent relaxation as a function of vascular smooth muscle cells, the SNP (a direct donor of NO) was used. There was no significant difference in vasorelaxation in response to SNP or in the sensitivity, as indicated by the LogEC50 values presented in the table below graph among the tested groups (Figure 2).

### 3.4. Effect of Carnosine Supplementation on Hypoxia-Induced Relaxation (HIR) of Aortic Rings

**Figure 3:**
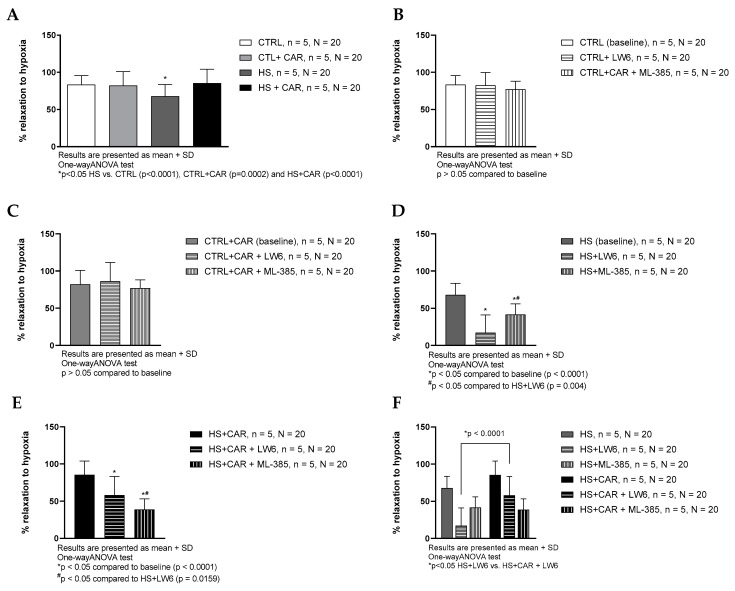
Hypoxia-induced dilation (HID) response of isolated rat aorta rings in CTRL, CTRL + CAR, HS, and HS + CAR groups without (**A**) and with HIF-1α inhibitor—LW6 and NRF2 inhibitor—ML-385 in CTRL (**B**), CTRL + CAR (**C**), HS (**D**), and HS + CAR (**E**) groups. Panel (**F**) presents comparison of the effect of HIF-1 alpha and NRF2 inhibition in HS and HS + CAR groups. Results are presented as mean + SD; n—number of rats, N—number of aortic rings; *^#^ *p* < 0.05, one-way ANOVA test.

The HS group exhibited significantly decreased relaxation in response to reduced pO_2_ (hypoxia-induced relaxation, HIR) compared to all the other groups of rats. The HS + CAR group exhibited significantly stronger HIR compared to the HS group of rats, to the levels similar to the CTRL and CTRL + CAR groups. Carnosine supplementation did not alter HIR in the CTRL group (Figure 3A). LW6 (HIF-1α inhibitor) and ML-385 (NRF2 inhibitor) did not affect HIR in the CTRL and CTRL + CAR groups (Figure 3B,C). HIR was significantly decreased in the presence of ML-385 or LW6 inhibitors in the HS and HS + CAR groups compared to the baseline in the respective groups (Figure 3D,E). LW6 decreased HIR to a greater degree in the HS group compared to the HS + CAR group (Figure 3F).

### 3.5. Effect of Carnosine Supplementation on Oxidative Stress, Antioxidative Capacity, and Superoxide Dismutase Activity

The results of the oxidative stress level, assessed by advanced oxidized protein products measurements (AOPPs); antioxidative capacity, assessed by the Ferric Reducing Antioxidant Power (FRAP) assay; and SOD enzyme activity are shown in Table 3. The serum AOPP concentration was significantly higher in the HS group compared to all other groups. Carnosine supplementation significantly decreased AOPPs in HS rats to levels similar to the CTRL group.

The HS group had a significantly lower level of antioxidant capacity compared to all other groups. The highest level of antioxidant capacity was determined in the CTRL + CAR group of rats. Carnosine supplementation increased FRAP in rats on an HS diet (HS + CAR group) compared to the HS group.

Superoxide dismutase (SOD) activity in serum samples was increased in the HS + CAR group compared to the other tested groups.

### 3.6. Effect of Carnosine Supplementation on Gene Expression in Aortic Tissue

#### 3.6.1. Antioxidant Enzymes’ mRNA Expression in Aortic Tissue

The relative mRNA expression of *SOD1* and *SOD2* was significantly increased in the CTRL + CAR and HS + CAR groups compared to the CTRL group and HS group, respectively. The relative expressions of *SOD3* and *CAT* were similar in all groups of rats. Carnosine supplementation significantly increased the relative expression of *GPx1* in the HS + CAR group compared to the CTRL and HS groups, while there was no significant difference between the CTRL+ CAR and HS + CAR groups. The relative expression of *GPx4* was significantly increased only in the HS +CAR group compared to the HS group (Table 4).

#### 3.6.2. Effect of Carnosine Supplementation on mRNA Expression of Enzymes Involved in Vascular Reactivity—Cyclooxygenases 1 and 2 (*COX1* and *2*) and Nitric Oxide Synthases (Inducible—*iNOS*, Endothelial—*eNOS*)

The results showed that there was no significant difference in the expression of *COX-1*, *COX-2*, and *iNOS* enzyme genes among groups. Gene expression of *eNOS* was significantly increased in the HS + CAR group compared to the HS group and compared to the CTRL group (Table 5).

#### 3.6.3. Effect of Carnosine Supplementation on mRNA Expression of *HIF-1α*, *NRF2*, and *NQO1*

mRNA expression of *HIF-1α* was similar among groups. *NQO1* gene expression was significantly increased in the CTRL + CAR group compared to the CTRL group, and in the HS + CAR group compared to the HS group and in the HS + CAR group compared to the CTRL. The mRNA expression of *NRF2* was significantly increased in the CTRL + CAR group compared to the CTRL and in the HS + CAR group compared to the CTRL and HS groups (Table 6).

## 4. Discussion

The salient findings of the present study are (a) peroral carnosine supplementation preserved endothelium-dependent relaxation (in response to ACh and reduced pO_2_) of isolated aortic rings of rats on an HS diet; (b) carnosine supplementation decreased markers of oxidative stress and increased antioxidant capacity (FRAP in blood and antioxidative enzymes in aortic tissue) in rats consuming an HS diet; (c) the effects of carnosine on the relaxation possibly occur via the HIF-1 alpha and NRF2 transcription factors and their down-stream targets, *eNOS* and *NQO1* genes, respectively.

Our study and other previous studies have shown altered vascular reactivity after an HS diet due to increased oxidative stress [1,2,3,5,13,36]. We have previously shown that an acute HS diet leads to impaired vasodilation of MCAs in response to an increase in blood flow due to a significant decrease in *GPx4* and *iNOS* gene expression and increased systemic and vascular oxidative stress [2]. In addition, the mechanisms underlying flow-induced dilation (FID) are influenced by dietary salt intake. In the low-salt (LS) group, FID was influenced by nitric oxide (NO), prostaglandins, and epoxyeicosatrienoic acids (EETs), whereas FID was reduced in the high-salt (HS) group and depended mainly on NO. In the HS group supplemented with TEMPOL, FID was restored and EETs could also contribute to FID, suggesting that oxidative stress—which is altered by HS or TEMPOL intake—may affect arachidonic acid metabolism, shifting the production of its metabolites and highlighting the role of oxidative stress in regulating FID. The impaired endothelium-dependent dilation and decreased NO bioavailability observed in the HS group were primarily due to increased endothelial concentrations of superoxide and other reactive oxygen species (ROS) [5]. Further, an HS diet alters the protein expression of the transcription factor HIF-1α, which may contribute to changes in the gene and protein expression of enzymes related to oxidative stress in the vascular system [5,36,37,38].

We demonstrated previously that oxidative stress reduced sensitivity to various vasodilatory stimuli in general [2,27], which occurred prior to the onset of elevated blood pressure after HS intake [2]. It is very important to emphasize that reduced NO is strongly associated with increased levels of ROS produced by NAD(P)H oxidase, xanthine oxidase, or unbound endothelial NO synthase within the vascular wall. In the present study, the addition of carnosine to an HS diet increased the gene expression of *eNOS* but also *NQO1*, thus preserving vasorelaxation in response to ACh. Hypoxia-induced relaxation (HIR) was impaired in the HS group compared to the other groups, and this impairment was prevented by carnosine supplementation, too, suggesting the role of oxidative stress in attenuated response to reduced pO_2_. Physiologically, a significant portion of the vasodilation response to hypoxia is driven by the activation of cyclooxygenase (COX), leading to the production of prostacyclin (PGI2), and subsequent activation of K_ATP_ channels [39]. Matarogui et al. showed that high concentrations of salt, together with COX-2 inhibition, reduced artery dilation [40]. According to their research, the elimination of oxidative stress does not affect the expression of the *COX-1* and *COX-2* genes [40], which aligns with the results of this study, where no significant changes in the expression of these genes were observed. In another study, attenuated vasodilation of middle cerebral arteries in response to reduced pO_2_ was mediated by NO in rats fed an HS diet [41], in agreement with the finding in the present study.

A considerable number of studies confirmed that carnosine contained important properties for defending the body against oxidative stress [8,42]. Carnosine acts through a direct antioxidant mechanism; due to its unique combination of antioxidant and antiglycation properties, it can alleviate cellular oxidative stress and inhibit the intracellular formation of reactive oxygen species (ROS) and reactive nitrogen species (RNS) [16]. To our knowledge, the antioxidant effect of carnosine on blood vessels following HS intake has not been studied yet. The present study showed that peroral carnosine supplementation prevented attenuation of both ACh-IR and HIR in rats on an HS diet. An HS diet did not affect the endothelium-independent vasorelaxation and the function of smooth muscle cells, consistent with our previous results [2,5,13]. Our results showed significantly decreased relaxation of aortic rings in HS + CAR rats in the presence of NRF2 or HIF-1α inhibitors, with the effect being more prominent after NRF2 inhibition in the HS + CAR rat group. On the other hand, the expression of the *NQO1* enzyme and the *NRF2* transcription factor in the CAR groups was significantly higher in the control and HS groups receiving carnosine (CTRL + CAR and HS + CAR) compared to their respective control groups, suggesting that carnosine can affect the expression of *NRF2*, which subsequently increases the expression of its target gene *NQO1*, thus increasing the defence mechanisms against oxidative stress. Previous studies have shown that an HS diet increases oxidative stress levels, which may have prompted the NRF2 transcription factor to translocate from the cytoplasm to the nucleus, where it activated the expression of a range of genes, including *NQO1*. It can be assumed that in the group exposed to an HS diet, oxidative stress increased, hindering normal cellular function, preventing the cells from defending themselves through the transcription of genes typically used to combat oxidative stress. Since oxidative stress impairs vasodilation, alteration of the NRF2 pathway by an HS diet may be a potential crucial step in endothelial damage and the proposed use of carnosine could be useful in pathologies associated with HS intake (e.g., atherosclerosis, high blood pressure). Clinical studies have investigated the therapeutic potential of carnosine for treating conditions such as diabetes, neurological disorders, and aging [43,44,45]. Importantly, studies on the role of carnosine in kidney diseases have shown protective properties, especially in acute kidney failure caused by ischemia, diabetic nephropathy, nephrotoxicity, and blood pressure regulation [45].

AOPPs originate from oxidatively modified albumin (its aggregates or fragments), but also from fibrinogen and lipoproteins. They promote oxidative stress and inflammation, thus participating in many pathophysiological processes of various diseases including Parkinson’s disease, multiple sclerosis, and others [46]. Previous studies have shown that AOPPs can increase the formation of ROS and the secretion of cytokines, thereby leading to endothelial cell damage and the development of endothelial dysfunction [12,47], as observed with an HS diet in the present study and a previous study [13]. AOPPs may be considered as novel and sensitive indicators of oxidative stress, since their formation is irreversible [48]. Our results showed a significantly elevated concentration of AOPPs in the serum of rats on an HS diet compared to other groups while AOPPs were normal after an HS diet with concomitant carnosine supplementation (HS + CAR), speaking in favour of carnosine’s beneficial effect.

Furthermore, carnosine supplementation preserved the antioxidative capacity in serum while HS per se decreased it. In addition, the present study demonstrated a significant increase in the vascular expression of *SOD1*, *SOD2, GPx1*, and *GPx4* in the rats that received carnosine supplementation during an HS diet. This agrees with findings in different animal models of oxidative stress. For example, the cardiac tissue of female Sprague Dawley rats consuming carnosine showed sustained SOD and GPx activity and prevented inactivation of CAT in doxorubicin toxicity studies [49] or in mouse models of acetaminophen-induced liver damage [50]. However, not all studies achieved the expected effect. For example, Stefani et al. did not show a significant effect on the levels of antioxidant enzymes in Wistar rats after cardiac arrest, although a favourable effect on lipid peroxidation levels and an anti-inflammatory effect were noted [51]. However, the absence of significant changes in antioxidant enzyme levels observed in these studies could occur due to the long delay in carnosine administration after the injury.

DNA microarray analyses have revealed that more than 2% of all human genes in arterial endothelial cells are regulated, either directly or indirectly, by HIF-1α including genes involved in vascular reactivity and structural adaptations [52,53]. For instance, under hypoxic conditions, such as haemorrhagic shock, HIF-1α modulates the expression of *eNOS*, *iNOS*, *HO-1*, *COX-2*, and influences the production of nitric oxide (NO) and prostaglandins [54]. In our previous studies, we have demonstrated that HS intake increases HIF-1α protein expression [5], primarily due to elevated reactive oxygen species (ROS) production [5,13]. During hypoxia, HIF-1α translocates to the nucleus, where it heterodimerizes with the β subunit to form the functional HIF-1 complex [55]. Notably, the generation of free radicals is essential for the expression of HIF-1α [56,57]. In the present study, inhibition of HIF-1α rendered significantly lower relaxation of HIR in the HS group compared to the HS + CAR group, suggesting that indeed in a condition of elevated levels of oxidative stress, such as an HS diet, the HIF-1α signalling pathway is crucial for maintaining relaxation in response to reduced pO_2_, which subsequently leads to the vascular reactivity impairments observed. However, carnosine supplementation reverses this effect, which can be related to the NRF2 transcription factor.

The limitation of the present study is a lack of changes in gene expression of *HIF-1*α and target genes in the condition of the HS diet. This may be explained by the cross-sectional study design, with one-time point sampling and measurements, since genetic changes may occur faster than functional response to pharmacological stimuli (i.e., inhibitors). However, previously we showed increased protein expression of HIF-1α in brain blood vessels after the same protocol of HS intake for 7 days in the same experimental model, which is related to the increased oxidative stress following HS intake [5,13]. We have not measured biochemical parameters and we have not performed expression analysis of ROS production in the aortic endothelial cell samples in this study due to technical constraints. However, we have just published an article showing that in cultured human endothelial cells (HAECs), increasing the NaCl concentration in medium to levels similar to those in the present animal studies leads to increased production of ROS [58]; these data support our current conclusions. It is important to emphasize that the results are analysed as a group and not divided by sex, since sex-related differences were not the scope of the present study, so it would be valuable to test the sex differences prior to translating the results to human biology.

## 5. Conclusions

Altogether, the results clearly support the hypothesis that carnosine affects the antioxidant status regulation by modifying expression of *NRF2* and its target genes, including antioxidative enzymes, leading to a decrease in oxidative stress and preservation of vasorelaxation in condition of an HS dietary intake.

## Figures and Tables

**Table 1:** List of primers and nucleotide sequences of determined mRNA genes’ expression.

Gene	Nucleotide Sequence
*SOD1*	forward: 5′-CTTCTGTCGTCTCCTTGCTTT-3′
	reverse: 5′-AGTGGTACAGCCTTGTGTATTG-3′
*SOD2*	forward 5′-GCGACCTACGTGAACAATCT-3′
	reverse 5′-AGCGACCTTGCTCCTTATTG-3′
*SOD3*	forward 5′-TGTTCTGCAACCTGCTACTG-3′
	reverse 5′-CATCCAGATCTCCAGGTCTTTG-3′
*GPx1*	forward 5′-TCCAGTATGTGTGCTGCTCG-3′
	reverse 5′-GTGTCCGAACTGATTGCACG-3′
*GPx4*	forward 5′-ATACGCCGAGTGTGGTTTAC-3′
	reverse 5′-GGCTGCAAACTCCTTGATTTC-3′
*CAT*	forward 5′-CTCAGGTGCGGACATTCTATAC-3′
	reverse 5′-CATTCTTAGGCTTCTGGGAGTT-3′
*COX1*	forward 5′-TCCTGTTGCGAGCCCAGTT-3′
	reverse 5′-GCCAGTGATAGAGGTGGTTGAAT-3′
*COX2*	forward 5′-GAAAGAAATGGCTGCAGAGTTGA-3′
	reverse 5′-GCAGGGCGGGATACAGTTC-3′
*HIF-1 alpha*	forward 5′-GCCCAGTGAGAAAGGGGA-3′
	reverse 5′-CGGCTGGTTACTGCTGGT-3′
*NQO1*	forward 5′-AGTGGAAACCCACGAAGCCTACAA-3′
	reverse 5′-TGAACCAGTACAGCGGGAACTGAA-3′
*NRF2*	forward 5′-AAGCCATTCACTCTCTGAACTTCT-3′
	reverse 5′-CTGGGACTTGTGTTTAGTGAAATG-3′
*eNOS*	forward 5′- CGAACAGCAGGAGCTAGAGG-3′
	reverse 5′- GAGGTGGATCTCTCCTGGT-3′
*iNOS*	forward 5′-TGGTGAGGGGACTGGACTTT-3′
	reverse 5′-CCAACTCTGCTGTTCTCCGT-3′
*β-actin*	forward 5′-TCTGTGTGGATTGGTGGCTCTA-3′
	reverse 5′-CTGCTTGCTGATCCACATCTG-3′

**Table 2:** Measurements of body mass and arterial pressure of SD rats.

Experimental Group		Body Mass [g]	SBP [mmHg]	DBP [mmHg]	MAP [mmHg]
CTRL	Male (N = 7)	348.11 ± 22.89	129 ± 4	92 ± 5	104 ± 4
	Female (N = 9)	247.00 ± 16.61 *	117 ± 10 *	80 ± 6	93 ± 7 *
	Mean	297.56 ± 19.75	123 ± 7	86 ± 6	99 ± 6
CTRL + CAR	Male (N = 9)	360.56 ± 27.72	131 ± 6	95 ± 6	107 ± 5
	Female (N = 7)	241.00 ± 12.04 *	120 ± 3 *	79 ± 8	93 ± 6 *
	Mean	300.78 ± 19.88	126 ± 3	87 ± 7	100 ± 5
HS	Male (N = 11)	349.10 ± 21.43	131 ± 9	90 ± 8	104 ± 8
	Female (N = 5)	247.33 ± 25.33 *	119 ± 2 *	81 ± 10	94 ± 7 *
	Mean	298.22 ± 23.38	125 ± 6	86 ± 9	99 ± 7
HS + CAR	Male (N = 10)	356.50 ± 37.52	129 ± 5	88 ± 12	101 ± 9
	Female (N = 6)	234.70 ± 12.07 *	120 ± 2 *	84 ± 6	96 ± 4
	Mean	295.60 ± 24.79	125 ± 4	86 ± 9	99 ± 6

Results are shown as mean ± SD (standard deviation); CTRL (control group, 0.4% NaCl), HS (4% NaCl in the diet for 7 days), CTRL + CAR (oral carnosine supplementation, 150 mg/kg/day for 7 days), and HS + CAR group (HS diet and carnosine supplementation). SBP—systolic blood pressure; DBP—diastolic blood pressure; MAP—mean arterial pressure; N—number of animals. * *p* < 0.05, unpaired *t*-test for between-sex differences; one-way ANOVA test for group comparison.

**Table 3:** Oxidative stress and antioxidant capacity.

	CTRL	CTRL + CAR	HS	HS + CAR
AOPPs [nmol/mL]	15.91 ± 7.04 ^‡^	14.97 ± 5.67 ^‡^	44.26 ± 12.96 *^†^	11.43 ± 3.09 ^‡^
FRAP [mM Trolox]	0.08 ± 0.02 ^‡^	0.11 ± 0.01 ^‡^	0.03 ± 0.02 *^†^	0.07 ± 0.03 ^‡^
SOD [U/mgP]	12.39 ± 1.31	12.81 ± 0.76	11.49 ± 1.06	17.72 ± 2.21 *^†‡^

Results are shown as mean ± SD (standard deviation); CTRL (control group, 0.4% NaCl), HS (4% NaCl in the diet for 7 days), CTRL + CAR (oral carnosine supplementation, 150 mg/kg/day for 7 days), and HS + CAR group (HS diet and carnosine supplementation). AOPP—advanced oxidized protein products; FRAP—ferric reducing antioxidant power; SOD—superoxide dismutase. One-way ANOVA test—* *p* < 0.05 vs. CTRL; ^†^ vs. CTRL + CAR and ^‡^ vs. HS.

**Table 4:** Relative expression of *SOD1*, *2*, and *3*, *CAT*, and *GPx1* and *4* mRNAs in rat aortas.

	CTRL	CTRL + CAR	HS	HS + CAR
*SOD1*	0.064 ± 0.03	0.181 ± 0.07 *	0.074 ± 0.03 ^†^	0.262 ± 0.09 *^‡^
*SOD2*	0.062 ± 0.02	0.162 ± 0.08 *	0.085 ± 0.06 ^†^	0.226 ± 0.11 *^‡^
*SOD3*	3.140 ± 2.27	2.351 ± 1.32	2.174 ± 1.78	1.499 ± 0.64
*CAT*	1.355 ± 0.28	1.593 ± 0.34	1.730 ± 0.49	1.966 ± 1.17
*GPx1*	0.415 ± 0.38	0.713 ± 0.13	0.621 ± 0.20	1.063 ± 0.44 *^‡^
*GPx4*	0.762 ± 0.61	0.974 ± 0.25	0.681 ± 0.16	0.981 ± 0.26 ^‡^

Data are presented as mean ± SD (standard deviation), n = 6 (number of rats per group). *SOD1, 2,* and *3*—superoxide dismutase; *CAT*—catalase, *GPx1* and *4*—glutathione peroxidase. One-way ANOVA test—* *p* < 0.05 vs. CTRL; ^†^ vs. CTRL + CAR and ^‡^ vs. HS.

**Table 5:** Relative expression of *COX1* and *2, iNOS*, and *eNOS* mRNAs in rat aortas.

	CTRL	CTRL + CAR	HS	HS + CAR
*COX1*	4.107 ± 3.00	3.997 ± 2.47	4.185 ± 2.62	4.322 ± 1.61
*COX2*	2.468 ± 0.77	2.828 ± 0.48	1.954 ± 0.86	2.208 ± 0.93
*iNOS*	0.273 ± 0.28	0.569 ± 0.19	0.291 ± 0.16	0.352 ± 0.20
*eNOS*	0.119 ± 0.16	0.278 ± 0.33	0.113 ± 0.12	0.582 ± 0.27 *^‡^

Data are presented as mean ± SD (standard deviation), n = 6 (number of rats per group). *COX1* and *2*—cyclooxygenase; *iNOS*—inducible nitric oxide synthase; *eNOS*—endothelial nitric oxide synthase. One-way ANOVA test—* *p* < 0.05 vs. CTRL; ^‡^ vs. HS.

**Table 6:** Relative expression of *HIF-1α*, *NRF2*, and *NQO1* mRNAs in rat aortas.

	CTRL	CTRL + CAR	HS	HS + CAR
*HIF-1α*	1.412 ± 0.75	1.576 ± 0.44	1.082 ± 0.33	1.324 ± 0.57
*NRF2*	0.046 ± 0.06	0.498 ± 0.37 *	0.149 ± 0.08	0.536 ± 0.22 *^‡^
*NQO1*	0.275 ± 0.15	0.925 ± 0.43 *	0.571 ± 0.29	2.064 ± 1.53 *^‡^

Data are presented as mean ± SD (standard deviation), n = 6 (number of rats per group). *HIF-1α*—hypoxia-inducible factor-1 alpha; *NRF2*—nuclear factor erythroid 2-related factor 2; *NQO1*—NAD(P)H dehydrogenase (quinone 1). One-way ANOVA test—* *p* < 0.05 vs. CTRL; ^‡^ vs. HS.

## Data Availability

The original contributions presented in this study are included in the article. Further inquiries can be directed to the corresponding author(s).
